# A case of intra-articular ganglion cysts of the knee joint: correlation between arthroscopic and magnetic resonance imaging

**DOI:** 10.1186/s12880-016-0138-8

**Published:** 2016-05-04

**Authors:** Sayaka Kodaira, Takahito Nakajima, Ryosuke Takahashi, Shingo Moriya, Tomoyuki Nakagawa, Hidenori Ohtake, Yoshito Tsushima

**Affiliations:** Department of Diagnostic Radiology and Nuclear Medicine, 3-39-22, Showa, Maebashi, Gunma Japan; Zenshukai Hospital, 1381, Ninomiya, Maebashi, Gunma Japan

**Keywords:** Ganglion, MRI, Arthroscopy, Intra-articular, knee

## Abstract

**Background:**

Intra-articular ganglion cysts of the knee are rare. Here we report a case of an arthroscopically confirmed ganglion cyst arising from the posterior cruciate ligament (PCL) along with preoperative magnetic resonance imaging (MRI) findings.

**Case presentation:**

A 39-year-old female admitted a hospital with left knee pain with flexion and extension. MRI revealed a cystic lesion along the PCL. The lesion exhibited slight but homogeneous hyperintensity on T1 weighted images. Thin septals were visible within the lesion. Arthroscopic examination revealed a mass lesion with a white fibrous capsule, located near the PCL. A gel-like liquid spurted from the mass upon puncture. The lesion was completely resected. Histological examination revealed loose connective tissue and fibroblasts with collagen, thus confirming the diagnosis of a ganglion cyst.

**Conclusion:**

Many reports have suggested intra-articular ganglion cysts of the knee are rare. In our study, a cystic lesion may have been impinged between the PCL and intercondylar notch, resulting in flexion and extension difficulty in the left knee. Arthroscopic resection is the major treatment of intra-articular ganglion cyst, and preoperative MRI findings can predict the correct arthroscopic approach. We have reported a case in which an intra-articular ganglion attached to the PCL.

## Background

Intra-articular ganglion cysts are well known to occur in the dorsal wrist, palm, and shoulder but rarely to originate in the knee joint [1, 2]. Ganglion cysts of the knee arise from both the cruciate ligaments and menisci as well as from the popliteal tendon, alar folds, and subchondral bone [2–4]. Here we report a case of an arthroscopically confirmed ganglion cyst arising from the posterior cruciate ligament (PCL) along with preoperative magnetic resonance imaging (MRI) findings.

## Case presentation

A 39-year-old female presented with a 1-year history of gait difficulty and left knee pain with flexion and extension. She also indicated listlessness and stiffness of her left lower leg; however, she had no history of trauma to the knee. Upon examination, the active range of motion of the left knee was slightly limited to 0°–120°.

**Fig. 1:**
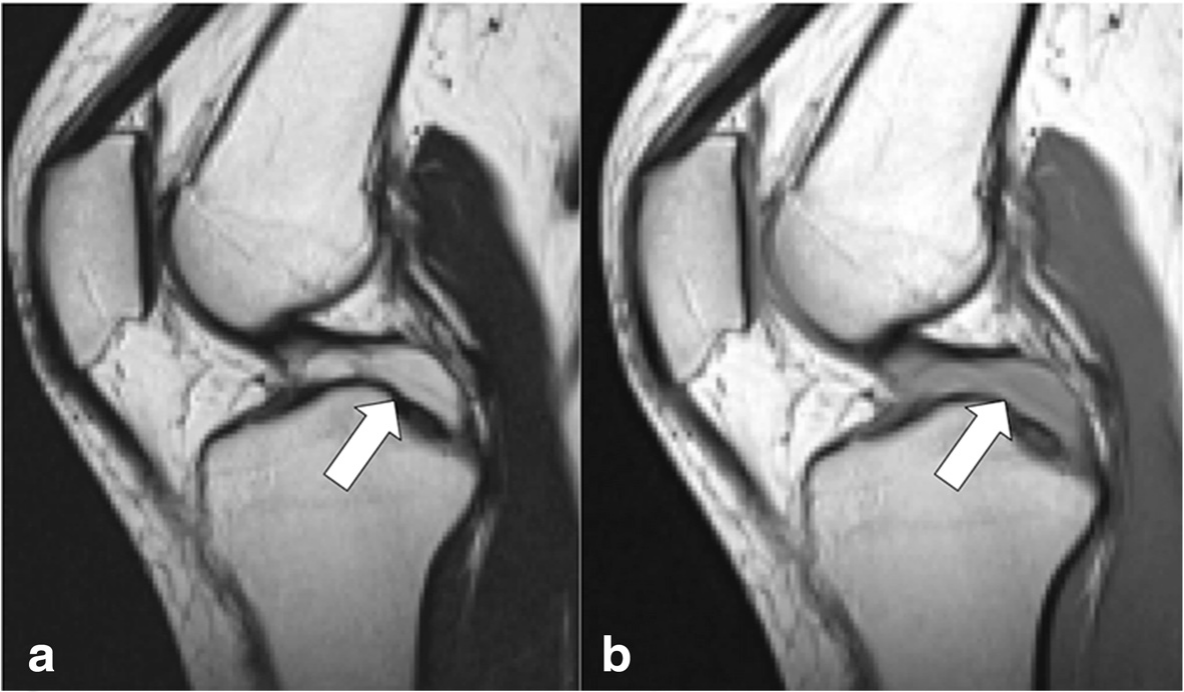
Sagittal magnetic resonance images revealed a cystic lesion located between the posterior cruciate ligament and intercondylar notch. This lesion exhibited apparent hyperintensity on T2 weighted images (T2WI, **a**) and slight hyperintensity on T1WI (**b**)

**Fig. 2:**
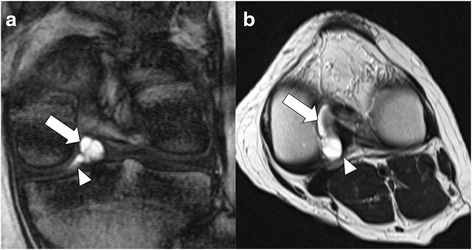
A multiloculated cystic lesion (arrow) was observed to be attached to the posterior cruciate ligament adjacent to lateral condyle of the femur on T2* weighted images (T2*WI, **a**) and T2WI (**b**). A small amount of thin wedge-shaped synovial fluid (arrowhead) was visible in the lateral joint space

MRI revealed a well-defined cystic lesion extending along the PCL, adjacent to the lateral condyle of the femur. The lesion exhibited slight but homogeneous hyperintensity, relative to the muscles on T1 weighted images and apparent hyperintensity on T2 weighted images. The lesion appeared to attach broadly to the PCL (Fig. 1). Thin septals were visible within the lesion, not containing a solid component. A small amount of thin wedge-shaped synovial fluid was observed in the lateral joint space, which was continuous with the lesion (Fig. 2). The menisci, cartilages, muscles, and ligaments were normal.

**Fig. 3:**
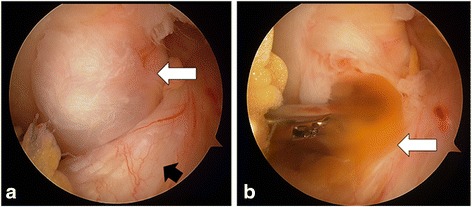
**a** Arthroscopic examination identified a mass located anteriorly to the posterior cruciate ligament (PCL). The cyst (white arrow) was located between the anterior cruciate ligament (black arrow) and PCL, tightly attached to the PCL, and covered with a white capsule. **b** The mass was arthroscopically excised in a piecemeal manner and the exuded viscous liquid (arrow) when punctured

Arthroscopic examination revealed a mass lesion with a white fibrous capsule, located near the PCL (Fig. 3a). A transparent dark reddish-brown viscous gel-like liquid spurted from the mass upon puncture with a probe (Fig. 3b). The lesion was completely resected in a piecemeal manner.

**Fig. 4:**
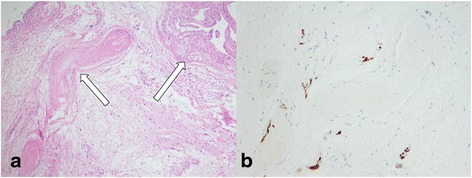
**a** Hematoxylin–eosin staining revealed loose connective tissue and fibroblasts with interspersed thicker bundles of collagen (10× magnification). **b** Immunohistochemistry revealed scattered S-100-positive cells among the loose connective tissue (40× magnification)

Histological examination revealed loose connective tissue and fibroblasts with interspersed thicker bundles of collagen, thus confirming the diagnosis of a ganglion cyst (Fig. 4a). Immunohistochemical analysis revealed scattered S-100-positive cells among the loose connective tissue (Fig. 4b).

No recurrence was detected for 6 months after successful arthroscopic excision.

## Discussion

García et al. first described preoperative MRI findings of cystic masses of the knee in 1991 [5]. Although Lee et al. had used computed tomography (CT) to evaluate cystic masses of the knee in 1987 [2], the majority of the cysts observed were popliteal cysts; intra-articular ganglion cysts of the knee remain rare. Differential diagnoses of ganglion cysts may include pigmented villonodular synovitis, synovial chondromatosis, meniscal or parameniscal cysts, and synovial hemangioma [4]. Intra-articular ganglion cysts of the knee are majorly detected as incidental findings using MRI, with a reported prevalence between 0.9 [6] and 1.3 % [3].

In 1997, Liem et al. reported 23 cases of intra-articular ganglion cysts of the knee along with the classifications and frequencies of the originating sites, which included the infrapatellar fat pad, within the anterior cruciate ligament (ACL), posterior to the ACL, anterior to the PCL, posterior to the PCL, in the intercondylar notch, at the bony attachment of ACL, and even outside of the knee joint [3]. In our study, a cystic lesion located on anterior portion of the PCL may have been impinged between the PCL and intercondylar notch, resulting in flexion and extension difficulty in the left knee [1, 7].

Brown and Dandy has reported 95 % of patients they treated with arthroscopic total resection had no recurrence [8]. CT-guided aspiration has also been reported in some cases [7, 9]. However, because complete excision using ultrasound-, CT-, and arthroscopic-guided aspiration is not possible and has high rate of recurrence, arthroscopic resection should be recommended [10]. Furthermore, preoperative MRI findings can predict the correct arthroscopic approach through an anterolateral, posteromedial, or posterolateral portal [10–12].

## Conclusion

We have reported a case in which an intra-articular ganglion attached to the PCL was detected using preoperative MRI. MRI is useful for depicting ganglion features, including size and location. This modality may also facilitate preoperative decisions regarding the direction of the arthroscopic approach.

## Consent

Informed consent was obtained from the patient for publication of this case report and accompanying images. Availability of data and materials.

## Availability of data and materials

Data are available on request from the corresponding author.

## Abbreviations

ACL: anterior cruciate ligament
CT: computed tomography
MRI: magnetic resonance imaging
PCL: posterior cruciate ligament
